# Horizontal analysis and longitudinal cohort study of chronic renal failure correlates and cerebral small vessel disease relationship using peak width of skeletonized mean diffusivity

**DOI:** 10.3389/fneur.2024.1461258

**Published:** 2024-09-06

**Authors:** Dan Wang, Zheng Sun, Yuehua Li

**Affiliations:** Institute of Diagnostic and Interventional Radiology, Shanghai Sixth People’s Hospital Affiliated to Shanghai Jiao Tong University School of Medicine, Shanghai, China

**Keywords:** MRI, PSMD, chronic renal failure, white matter lesion, cerebrovascular

## Abstract

**Background and purpose:**

Peak width of skeletonized mean diffusivity (PSMD) is an MRI-based biomarker that may reflect white matter lesions (WML). PSMD is based on skeletonization of MR DTI data and histogram analysis. Both chronic renal failure (CRF) and WML may be affected by multisystemic small-vessel disorder. We aimed to explore the relationship between PSMD and estimated glomerular filtration rate (eGFR).

**Methods:**

Fifty followed-up CRF patients matched for age, sex, hypertension and smoking status were enrolled and classified into a progressive group (*n* = 16) and stable group (*n* = 34) based on eGFR levels. Longitudinal and horizontal differences of PSMD were compared between the progressive and stable groups at the initial and follow-up time points. Pearson’s correlation was used for correlation of eGFR with PSMD and WML (per Fazekas scale score). ROC was used to measure the sensitivity of PSMD and WML score to changes of eGFR.

**Results:**

At the follow-up time point, PSMD of the progressive group was significantly higher than at the initial time point (*p* < 0.001), and PSMD of the progressive group was significantly higher than stable group (*p* < 0.001). PSMD and eGFR were significantly correlated. AUC curves explored that ΔPSMD (PSMD changes at the follow-up and initial time points) and follow-up PSMD was better for the classification of progressive and stable groups.

**Conclusion:**

PSMD has significant correlation with eGFR, PSMD can reveal a close relationship between WML and CRF.

## Highlights

- PSMD is based on skeletonization of MR DTI data and histogram analysis.
- CRF and WML may be affected by multisystemic small-vessel disorder.
- To explore the relationship between PSMD and eGFR.

## Introduction

Chronic renal failure is often accompanied by nervous system pathology. The earliest chronic renal failure (CRF)-associated lesion occurring in the nervous system is damage to small blood vessels including small arteries, arterioles, capillaries, and small veins (1). These vessels have poor collateral anastomoses, causing increased ischemic susceptibility to the deep white matter of the central nervous system. White matter lesion (WML) is a part of cerebral small vessel disease (CSVD) (2–5).

The correlation between CRF and WML is mainly because of similar hemodynamics, and anatomical and functional features of the kidney and brain (4). Therefore, CRF-associated pathological changes in small vessels of the kidneys may also exist in the small arteries of the brain (6).

Recently, Baykara et al. (7–9) used a new imaging marker called the peak width of skeletonized mean diffusivity (PSMD) in the brain to assess CSVD. PSMD is based on DTI, white matter tract skeletonization, and histogram analysis (7). Skeletonization focuses the analysis of mean diffusivity (MD) on the main fiber tracts, thereby largely eliminating CSF contamination (7). Whole-brain histogram analysis is particularly appropriate when dealing with diffuse diseases and when quantifying total disease burden. In some studies, PSMD appears to be robust and promising for studies on WML in large populations (8, 10, 11).

PSMD has been provided to be related to cerebral small vessel diseases, especially sensitive to small vessel-related white matter abnormalities. However, despite the common nature and high prevalence of cerebrovascular disease in patients with renal failure, the potential application of PSMD in renal failure has scarcely been investigated.

In this study, we used PSMD to explore the progression of WML in CRF based on MR diffusion images, and the relationship of cerebral WML and CRF.

## Materials and methods

### Participants

This retrospective analysis, conducted between December 2015 and December 2019, involved a cohort of 50 patients diagnosed with chronic renal failure (CRF) primarily caused by glomerulonephritis, hypertension, and diabetes et al., leading to chronic kidney dysfunction. The inclusion criteria were based on the clinical diagnostic guidelines outlined by the Kidney Disease Outcomes Quality Initiative (KDOQI), requiring patients to meet the criteria for stage III or IV CRF. CRF is characterized by prolonged renal damage lasting for more than 3 months, along with various causes of chronic kidney dysfunction. These causes may manifest as abnormal blood or urine components, abnormal imaging results, or a persistent decrease in estimated glomerular filtration rate (eGFR) below 60 mL/min/1.73 m^2^ for more than 3 months.

**Figure 1:**
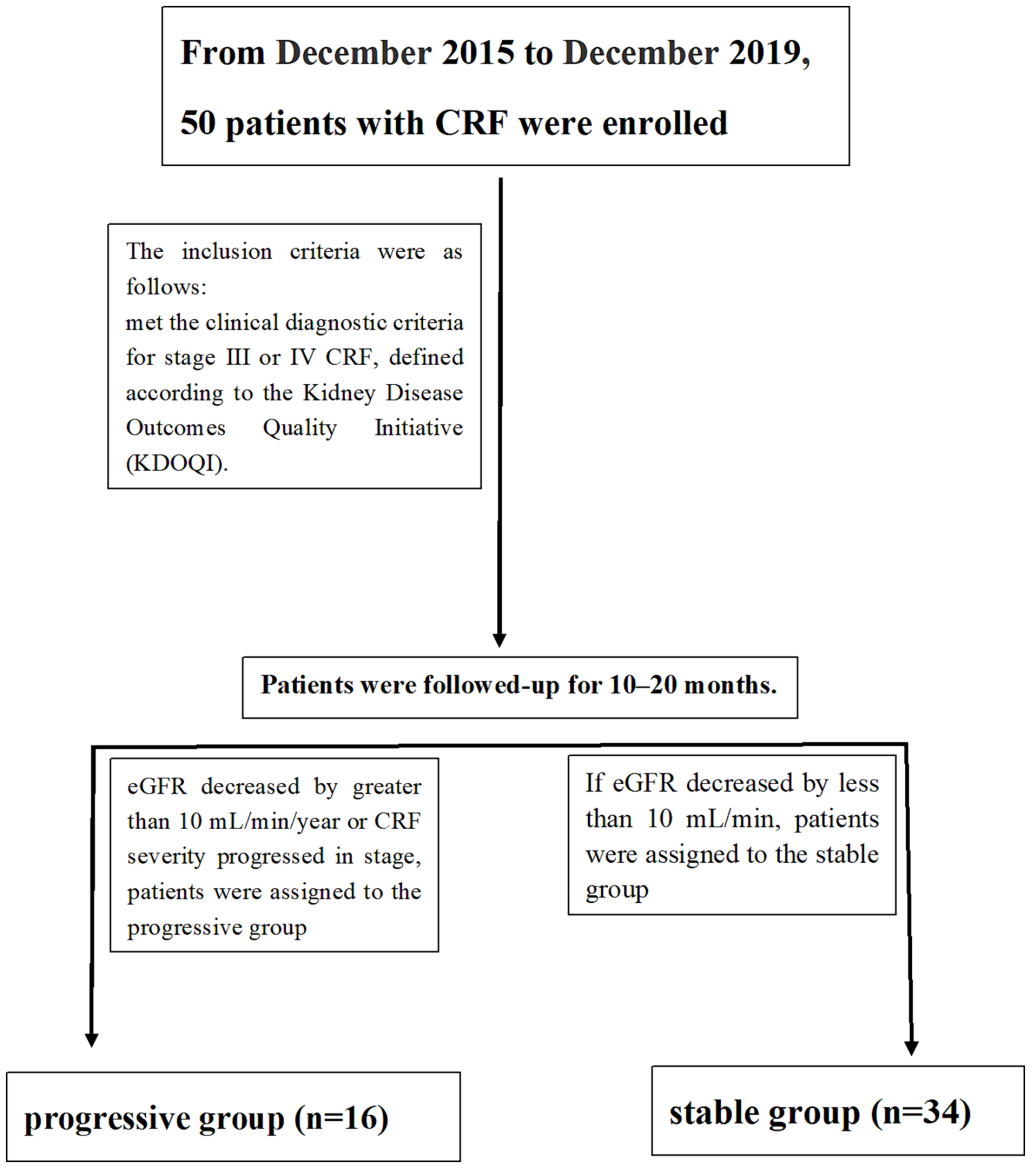
Flow chart.

The patients were followed up for a duration of 10 to 20 months. Those who exhibited a decline in eGFR greater than 10 mL/min/year or showed progression in CRF severity were classified into the progressive group (12). Conversely, patients with a decline in eGFR less than 10 mL/min were categorized into the stable group (Flow chart and data is showed in Figure 1 and Table 1).

**Table 1:** Clinical data for participants (progressive group and stable group).

	Progressive group	Stable group	Comparisons between two groups (*p*-value)
Number (*N*)	16	34	
Age (years)	52.35 ± 6.99	54.46 ± 8.73	
Sex (male/female)	11/5	19/15	0.39
Proportion who are smokers (y/n)	7/9	12/22	0.57
Hypertension (%)	4 (25%)	8 (23.53%)	0.91
Diabetes (type 2)	4 (25%)	9 (26.47%)	0.91
Total cholesterol (TC) (mmol/L)	4.87 + 0.93	5.29 + 0.97	0.15
Triglycerides (TG)	1.43 + 0.45	1.51 + 0.47	0.56
Fasting blood glucose (FBG)	5.41 + 0.48	5.63 + 0.45	0.13
Urine erythrocytes	2.44 + 1.46	1.65 + 1.5	0.09
Urine protein			
<0.2 g/L	*n* = 0	*n* = 2	
0.2 g to 1.0 g/L	*n* = 3	*n* = 7	
1.0 to 2.0 g/L	*n* = 4	*n* = 8	
2.0 to 4.0 g/L	*n* = 3	*n* = 12	
>4.0 g/L	*n* = 6	*n* = 5	
eGFR			
Initial eGFR	35.868 + 10.086	37.253 + 11.865	
Follow-up eGFR	23.28 + 8.10	36.95 + 11.05	
Horizontal comparisons between initial and follow up groups	^*^p < 0.001	*p* = 0.807	
Initial CKF stage			
Stage III	11	21	
Stage IV	5	13	
CKF stage at follow-up			
Stage III	3	21	
Stage IV	13	13	

^*^p < 0.05 indicated statistical significance. eGFR, estimated glomerular filtration rate; CRF, chronic renal failure.

### MR examination

All participants underwent MR examinations from December 2015 to December 2019. A 3 T MR scanner (MAGNETOM Verio, Siemens Healthcare, Erlangen, Germany) with a 32-channel head coil was used. The imaging sequences included conventional MR sequences (T1- and T2-weighted image) and diffusion tensor images (DTI). Protocols were as follows: DTI was performed for six *b*-values (0, 50, 500, 1,000, 1,500, 2,000, and 2,500 s/mm^2^) and 30 directions: FOV, 230 mm; TR/TE, 5100/109 ms; voxel size, 1.8 × 1.8 × 3 mm^3^; slice thickness, 3 mm; and number of slices: 32, DTI sequence takes 10 min 38 s.

#### Processing of PSMD

**Figure 2:**
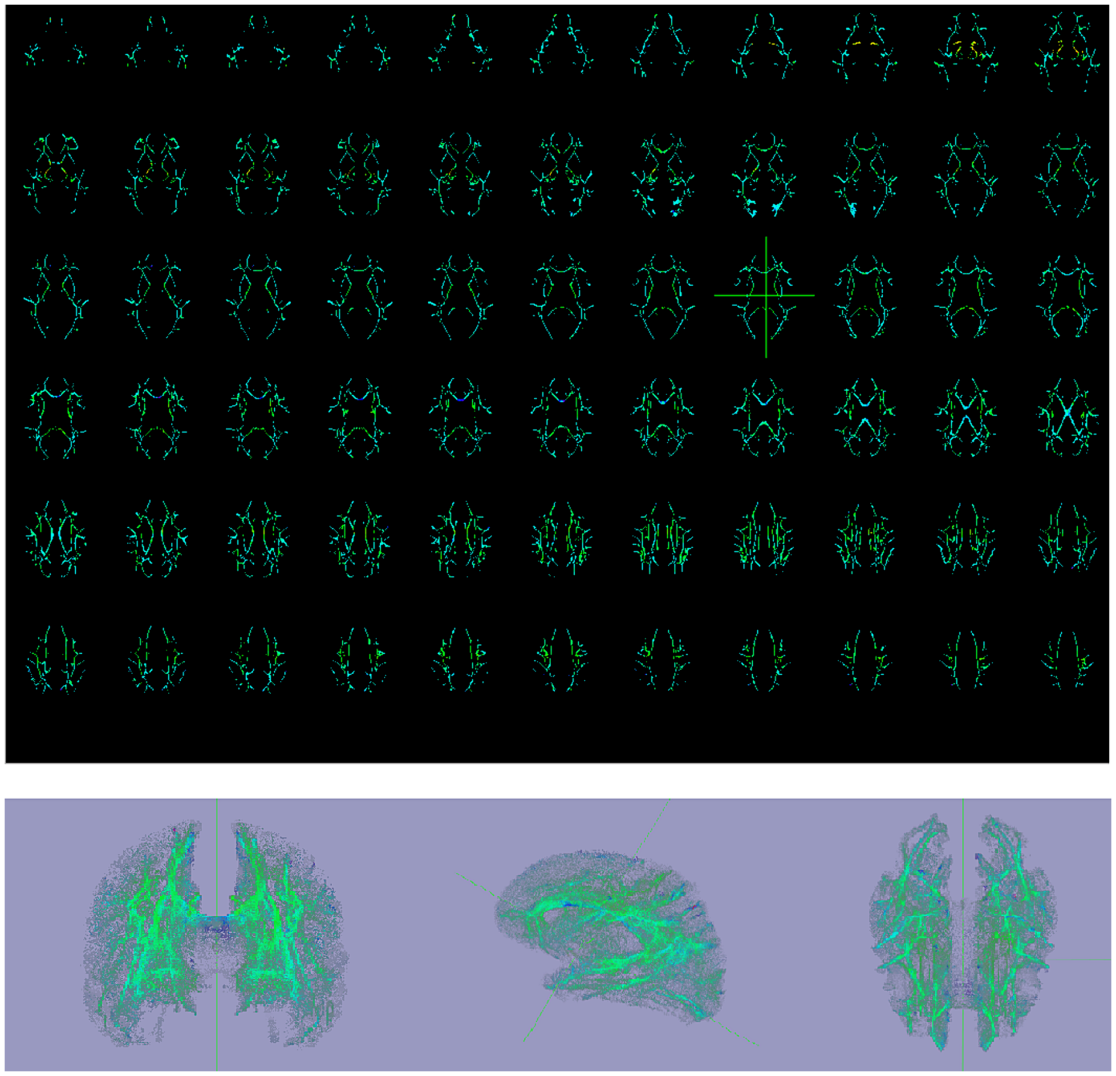
Initial MR examination, PSMD skeletonization images, and 3D PSMD skeletonization images of a 59-year-old patient in the progressive group. (1) PSMD skeletonization images were calculated, axial skeleton images of MD from top to base of the skull and 3D PSMD skeleton image. (2) 3D PSMD skeletonization images in axial, sagittal, and coronal orientations.

**Figure 3:**
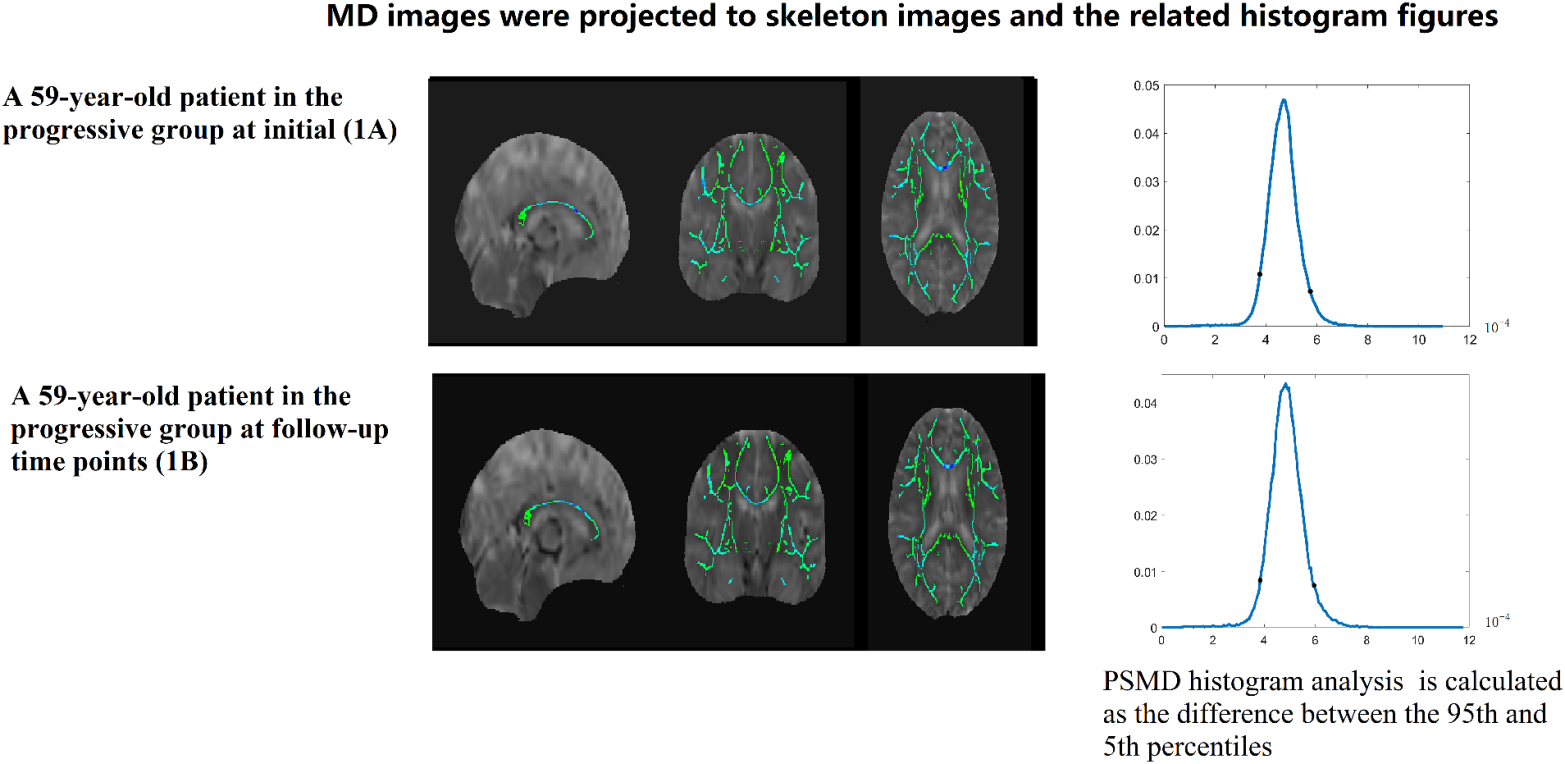
A 59-year-old patient in the progressive group at initial (1A) and follow-up time points (1B). MD images were projected to skeleton images and the related histogram figures. PSMD histogram analysis of the MD data is calculated as the difference between the 95th and 5th percentiles.

**Figure 4:**
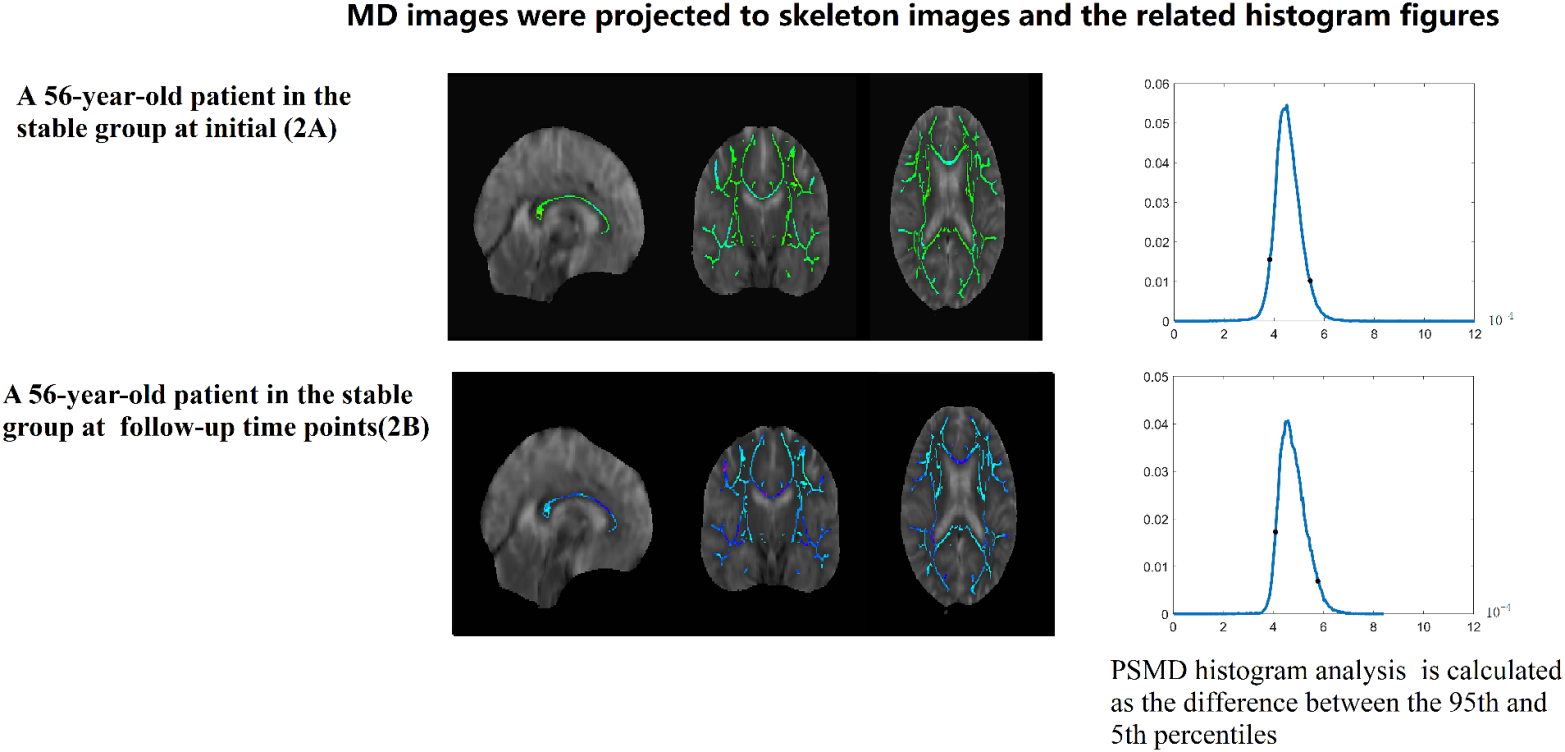
A 56-year-old patient in the stable group at initial (2A) and follow-up (2B) time points. MD images were projected to skeleton images and the related histogram figures. PSMD histogram analysis of the MD data is calculated as the difference between the 95th and 5th percentiles.

PSMD calculations include skeletonization of MR DTI data and histogram analysis Baykara et al. (7–9). (1) Preprocessing algorithms: include removing non-brain tissue and eddy current correction. (2) Skeletonization of MR DTI data: FA images of participants were registered to the FMRIB 1-mm standard space. Then, the FA data of each subject was projected onto the skeleton. According to the generated mapping matrix, the MD is projected onto the skeleton. Finally, the threshold was further set to reduce the influence of CSF on the MD skeleton. (3) Histogram analysis: PSMD is calculated as the difference between the 95th and 5th percentiles of the MD skeleton histogram analysis. All steps from (1) to (3) automatic calculation processes of PSMD follows the program described by Baykara et al. (7–9)^1^ (Data is showed in Figures 2–4).

#### Image analysis: WML Fazekas scale evaluation

One neuroradiologist with 9 years of experience was highly trained before reviewing images and recording WML locations and ratings.

A WML was defined as a signal abnormality of variable size in the white matter consisting of hyperintensity on FLAIR without cavitation and decreased signal on T1-weighted images, according to previous studies (2). Fazekas scale (0–3 points) was used as follows: grade 0 (normal), grade 1 (spot lesion), grade 2 (fusion lesion), and grade 3 (large fusion lesion) (13).

### Statistics

After passing the variance equality test, paired sample *t*-tests were used to compare the longitudinal differences in WML score and PSMD within the two groups (the comparison is the difference between initial and follow-up time for the progressive group and stable group). *p* < 0.05 was statistically significant. Two-sample *t*-test of WML and PSMD was used to compare the horizontal difference between the two groups at two time points (initial and follow-up). Covariance analysis was used to remove the influence of different follow-up time. Further, the ΔPSMD difference was also compared by covariance analysis, as was ΔWML (WML changes of follow-up and initial time point).

Pearson’s correlation was used for correlation of eGFR with PSMD and WML, and Pearson’s correlation was also used for correlation of ΔGFR with ΔPSMD and ΔWML.

AUC curves were plotted to explore which parameter is better for the classification of progressive group and stable group.

We conducted the statistical analysis using SPSS VERSION 27 software.

## Results

### Participants

In all, 50 patients with CRF were enrolled and classified into a progressive group (*n* = 16) and stable group (*n* = 34). There was no significant difference between the two groups with respect to age, sex, hypertension, diabetes, and smoking status. Covariance analysis was used to compare data between groups without the influence of follow-up time.

#### PSMD

##### Longitudinal comparison at different time points in the same group

**Figure 5:**
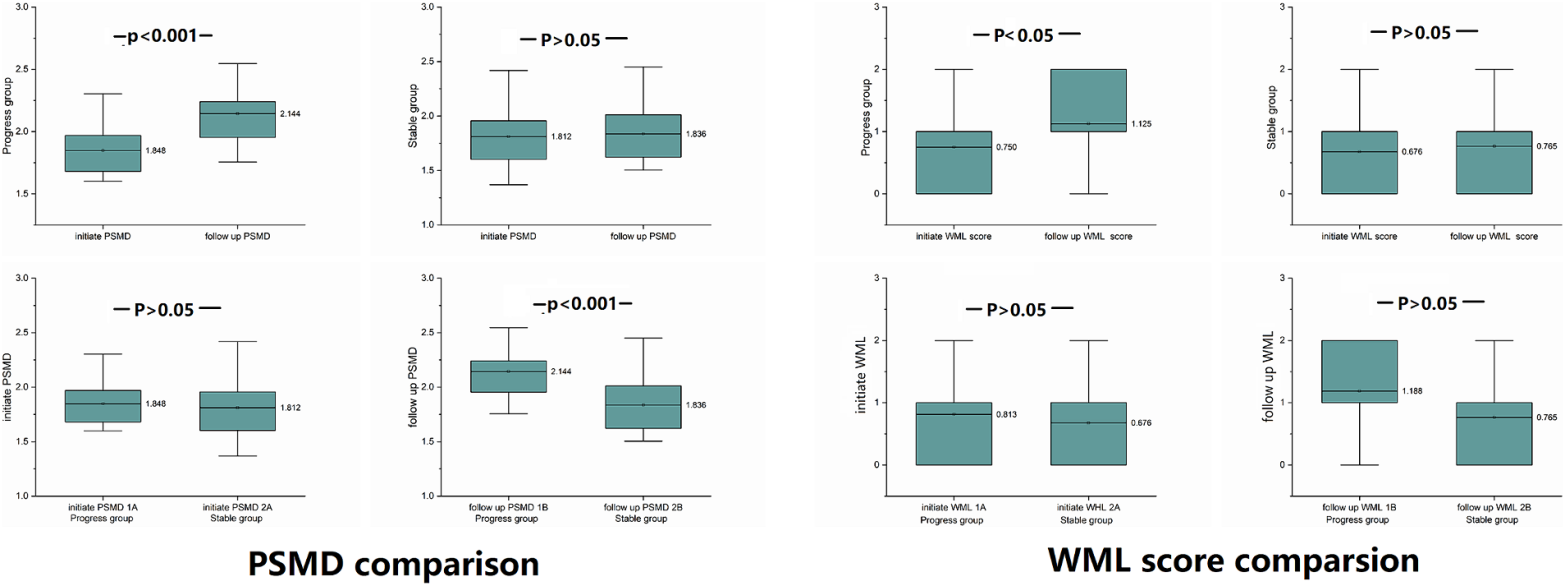
PSMD and WML differences were compared between the progressive and stable groups at the same time point, and comparison within groups at the initial and follow-up time points: at the follow-up time point, PSMD of the progressive group was significantly higher than at the initial time point (*p* < 0.001), and PSMD of the progressive group was significantly higher than that of the stable group (*p* < 0.001).

In the progressive group, PSMD at the follow-up time point was significantly higher than PSMD at the initial time point (*p* < 0.001). There is no significant difference between two time points of the stable group (*p* = 0.130) (Data is showed in Figure 5).

##### Horizontal comparison between the two groups at same time points

At the follow-up time point, the PSMD of progressive group was significantly higher than the PSMD of stable group (*p* < 0.001).

At the initial time point, there was no significant difference between the two groups (*p* = 0.629) (Data is showed in Figure 5).

##### ΔPSMD (PSMD changes of follow-up time point and the initial time point)

The PSMD of the progressive group showed significantly higher increase than the stable group (*p* < 0.001).

#### WML

Only WMLs in the progressive group were exacerbated, there was no significant difference between the two groups at the same time point.

##### Longitudinal comparison at different time points in the same group

WMLs in the progressive group were exacerbated at follow-up compared to the initial exam (*p* = 0.029).

There was no significant difference between the initial time point and follow-up time point of the stable group (*p* = 0.083) (Data is showed in Figure 5 and Table 2).

**Table 2:** Intra-group longitudinal comparison of PSMD, WML Fazekas scores and eGFR and at initial and after follow-up time points: [progressive group at initial exam (group 1A), progressive group at follow-up (group 1B), stable group at initial exam (group 2A), stable group at follow-up (group 2B)].

	Number	eGFR		ΔeGFR		PSMD		ΔPSMD		WML Fazekas scores		ΔWML Fazekas scores	
		Mean	SD	Mean	SD	Mean	SD	Mean	SD	Mean	SD	Mean	SD
Group 1A	16	35.868	10.086	−10.092	8.948	1.848	0.208	0.296	0.22	0.75	0.683	0.25	0.577
Group 1B	16	23.28	8.10			2.144	0.223			1.125	0.719		
Comparison 1A vs. 1B *p*-value		<0.001^*^				<0.001^*^				0.029			
Group 2A	34	37.253	11.865	−0.302	7.132	1.812	0.259	0.024	0.091	0.676	0.768	0.088	0.288
Group 2B	34	36.95	11.05			1.836	0.255			0.765	0.781		
Comparison 2A vs. 2B *p*-value		0.807				0.13				0.083			
Comparison group1 vs. group 2 *p*-value				<0.001^*^				<0.001^*^				0.032	

ΔPSMD (PSMD changes of follow-up time point and initial time point). ^*^p < 0.05 indicated statistical significance.

##### Horizontal comparison between the two groups at the same time points

At two time points, there was no significant difference between the two groups either at the initial time point (*p* = 0.544) or at the follow-up time point (*p* = 0.127) (Data is showed in Figure 5 and Table 3).

**Table 3:** Horizontal comparison of PSMD, WML Fazekas scores and eGFR between the progressive group and stable groups at the same time point [progressive group at initial exam (group 1A), progressive group at follow-up (group 1B), stable group at initial exam (group 2A), stable group at follow-up (group 2B)].

	Number	eGFR		PSMD		WML Fazekas scores	
		Mean	SD	Mean	SD	Mean	SD
Group 1A	16	35.868	10.086	1.848	0.208	0.813	0.655
Group 2A	34	37.253	11.865	1.812	0.259	0.676	0.768
Comparison between group 1A vs. 2A	*t* value	0.403		0.487		0.611	
	*p*-value	0.689		0.629		0.544	
Group 1B	16	23.28	8.10	2.144	0.223	1.188	0.655
Group 2B	34	36.95	11.05	1.836	0.255	0.765	0.781
Comparison between group 1B vs. 2B	*F* value	19.492		16.749		2.408	
	*p*-value	<0.001^*^		<0.001^*^		0.127	

^*^p < 0.05 indicated statistical significance.

##### ΔWML (WML changes to follow-up time point and initial time point)

ΔWML of progressive group significantly higher than stable group (*p* = 0.032).

###### Pearson’s correlation for eGFR with PSMD and WML

Initial PSMD with initial eGFR, follow-up PSMD with follow-up eGFR, PSMD with ÄGFR all significantly correlated respectively (Pearson correlation coefficients were −0.405, −0.445, and −0.438, respectively). Follow-up WML was significantly correlated to follow-up eGFR (Pearson correlation coefficient was 0.352) (Data is showed in Table 4).

**Table 4:** Pearson’s correlation for eGFR with PSMD and WML Fazekas scores, and Pearson’s correlation for ΔeGFR with ΔPSMD and ΔWML Fazekas scores.

Correlation coefficients	Initial GFR	Initial PSMD	Initial WML Fazekas scores	Follow-up PSMD	Follow-up WML Fazekas scores	ΔWML Fazekas scores score	ΔPSMD
Initial eGFR	Pearson corr.	−0.405	−0.223				
	*p*-value	0.004^*^	0.119				
Follow-up eGFR	Pearson corr.			−0.445	−0.352		
	*p*-value			0.001^*^	0.012^*^		
ΔeGFR	Pearson corr.					−0.225	−0.438
	*p*-value					0.117	0.001^*^

Two-tailed test of significance was used. ^*^Correlation is significant at the 0.05 level.

###### AUC curve results

**Figure 6:**
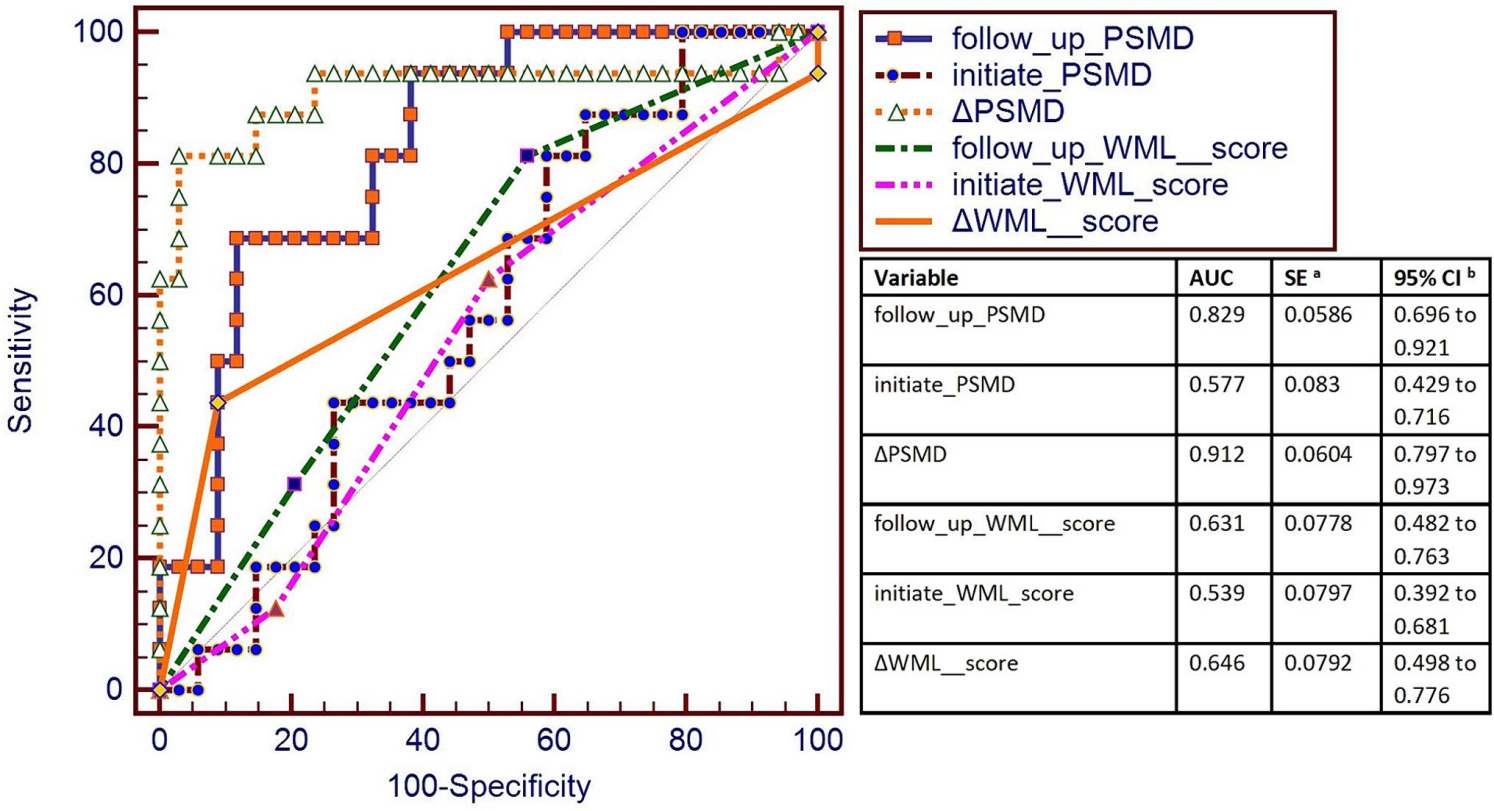
AUC was used to measure the sensitivity of PSMD and WML score to changes in eGFR. The highest AUC was of ΔPSMD (0.912), and the second-highest AUC was of the follow-up PSMD (0.829). AUC curve showed that ΔPSMD (PSMD changes of follow-up time point and the initial time point) and follow-up PSMD were better for the classification of progressive group and stable group.

The highest AUC is ΔPSMD (0.912), the second-highest AUC is follow-up PSMD (0.829) (Data is showed in Figure 6).

## Discussion

This is a study about using PSMD to study the relationship between renal failure and cerebral small vessel disease. Results of this study supported the stability of PSMD as an MR biomarker for cerebrovascular disease, especially sensitive to small vessel-related white matter abnormalities. This study found out that with deterioration of renal function, as reflected by decreased eGFR, the PSMD increases more in the progressive group than the stable group. PSMD is a more sensitive biomarker than WML score. These observations suggest that PSMD increased with CRF progressed.

PSMD has been proved to be a sensitive biomarker for small vessel disease (14–19). DTI images are more sensitive than conventional MRI T1W, T2W, and FLAIR images in detecting white matter changes. Histograms of MR parameter values measured in the whole brain are increasingly being used to characterize subtle disease that affects large parts of the brain. Based of DTI, PSMD uses MD skeletonization and histogram analysis, so it is advantageous in assessing the effect of CRF of cerebral small vessel white matter lesions (1). Studies have shown that histogram peak height measures were associated with cognitive function and CSVD (20).

### Chronic renal failure and PSMD and white matter lesions

Kidney hemodynamics are similar to that of brain, as both of their vascular beds have very low resistance and are sensitive to fluctuations in blood pressure. Moreover, it is widely accepted that chronic renal insufficiency is an independent risk factor for cerebrovascular disease (4, 21).

In this study, PSMD results support previous studies that PSMD is sensitive to small vessel-related white matter abnormalities (9). PSMD showed higher AUC than WML Fazekas scale scores, indicating that PSMD is a more sensitive MR biomarker to detect white matter lesion. Additionally, at the follow-up time point, PSMD detected worse white matter lesion in the progressive than stable group. eGFR is a reliable indicator of renal function, and is commonly used clinically to assess and monitor kidney function.

### Relationship of PSMD and renal function

The results of the this study are consistent with these conclusions, specifically that declining renal function, the greater the degree of white matter degeneration, the higher the PSMD value (4, 20, 21).

### The clinical application of PSMD

CSVD is increasingly recognized as a significant contributor to vascular cognitive disorders, including mild cognitive impairment and vascular dementia. The early detection of CSVD is pivotal for initiating targeted interventions that may include pharmacological treatments, lifestyle modifications, and cognitive training. These interventions are essential for mitigating the progression of cognitive impairment. Moreover, to deepen the understanding of the linkages between CSVD and CKD and explore novel diagnostic and therapeutic modalities. Collectively, these efforts contribute to the advancement of patient care and the overall improvement of clinical outcomes in the management of CKD and CSVD.

## Conclusion

This study is a first attempt to try to explore the progression of WML in CRF with PSMD. Previously, PSMD technology was rarely used to study the situation of cerebral small vessel disease in renal failure patients. This study revealed two important findings. Firstly, this study certificated that PSMD is closely related to eGFR and CRF. This study expands the understanding of the link between cerebral small vessel disease and renal failure. Secondly, there is bold speculation that when white matter lesions progress, renal function in patients with CRF may correspondingly decline. Conversely, when CRF declines, CSVD may also progress and is require more careful attention of physicians and patients. But imitated to the numbers of participants and follow-up time, these speculations require further validation in future large-sampled studies.

## Data Availability

The original contributions presented in the study are included in the article/supplementary material, further inquiries can be directed to the corresponding author.

## Footnotes

^1^ http://www.psmd-marker.com
